# Overexpression of the DEAD-Box RNA Helicase Gene *AtRH17* Confers Tolerance to Salt Stress in *Arabidopsis*

**DOI:** 10.3390/ijms19123777

**Published:** 2018-11-28

**Authors:** Linh Vu Nguyen, Hye-Yeon Seok, Dong-Hyuk Woo, Sun-Young Lee, Yong-Hwan Moon

**Affiliations:** Department of Integrated Biological Sciences, Pusan National University, Busan 46241, Korea; nhqdkr@gmail.com (L.V.N.); peachworld6@gmail.com (H.-Y.S.); humblewoo@hanmail.net (D.-H.W.); symoonlee@gmail.com (S.-Y.L.)

**Keywords:** activation tagging line, *Arabidopsis*, AtRH17, DEAD-box RNA helicase, overexpression, salt stress

## Abstract

Plants adapt to abiotic stresses by complex mechanisms involving various stress-responsive genes. Here, we identified a DEAD-box RNA helicase (RH) gene, *AtRH17*, in *Arabidopsis*, involved in salt-stress responses using activation tagging, a useful technique for isolating novel stress-responsive genes. AT895, an activation tagging line, was more tolerant than wild type (WT) under NaCl treatment during germination and seedling development, and *AtRH17* was activated in AT895. AtRH17 possesses nine well-conserved motifs of DEAD-box RHs, consisting of motifs Q, I, Ia, Ib, and II-VI. Although at least 12 orthologs of *AtRH17* have been found in various plant species, no paralog occurs in *Arabidopsis*. AtRH17 protein is subcellularily localized in the nucleus. *AtRH17*-overexpressing transgenic plants (OXs) were more tolerant to high concentrations of NaCl and LiCl compared with WT, but no differences from WT were detected among seedlings exposed to mannitol and freezing treatments. Moreover, in the mature plant stage, *AtRH17* OXs were also more tolerant to NaCl than WT, but not to drought, suggesting that *AtRH17* is involved specifically in the salt-stress response. Notably, transcriptions of well-known abscisic acid (ABA)-dependent and ABA-independent stress-response genes were similar or lower in *AtRH17* OXs than WT under salt-stress treatments. Taken together, our findings suggest that AtRH17, a nuclear DEAD-box RH protein, is involved in salt-stress tolerance, and that its overexpression confers salt-stress tolerance via a pathway other than the well-known ABA-dependent and ABA-independent pathways.

## 1. Introduction

Adverse abiotic stresses, such as high salinity, drought, extreme temperature, and excessive light limit growth, development, and productivity in plants. Plants show dynamic responses to adapt to those abiotic stresses at the biochemical, physiological, and molecular levels, thus enabling them to survive under variable abiotic stress conditions [1]. Adaptation to abiotic stresses requires coordinated modulations in plant metabolism, cell growth, division, and differentiation, all of which are dependent on gene control systems that are regulated by complex mechanisms [2].

RNA helicases (RHs) are ubiquitous proteins and essential conserved enzymes that function in both prokaryotes and eukaryotes [3,4]. RHs are divided into six superfamilies (SF1–SF6) depending on their specific motif sequences and domain structures, and SF2, the largest RH superfamily, mainly comprises the DEAD-box RHs [3,4]. In plants, 58 and 62 DEAD-box RHs have been identified in *Arabidopsis* (*Arabidopsis thaliana*) and in rice (*Oryza sativa*), respectively, to date [4,5,6,7].

DEAD-box RHs harbor nine conserved motifs (Q, I, Ia, Ib, and II-VI), and consist of 400–700 aa [3,8,9,10]. Motif Q regulates adenosine triphosphate (ATP) binding and hydrolysis; motif I (also known as the Walker A motif) is involved in the interaction between the ATP and Mg^2+^ ion; motif Ia forms a groove to bind to single-stranded DNA/RNA; motif II (otherwise known as the Walker B motif or DEAD motif) interacts with Mg^2+^ ion; motif III functions in NTPase and helicase activities and is responsible for RNA unwinding; and motif VI, part of ATP-binding cleft, is involved in helicase and NTPase activities. The molecular functions of motifs Ib, IV, and V remain unclear [4,5]. The “DEAD-box” designation is due to the presence of the conserved protein sequence Asp-Glu-Ala-Asp (D-E-A-D) in motif II [9,10,11], a sequence that is characteristic of DEAD-boxes. In addition to these conserved motifs, there are also N-terminal and C-terminal extended regions in each DEAD-box protein that vary widely in terms of their size and composition; it has been proposed that they function as substrate-binding specificities or as subcellular localization signals, or possibly that they interact with accessory components [12,13,14]. For example, a nucleoside triphosphate (NTP)-binding motif, which is required to function in an ATP-dependent manner, is found in all DEAD-box RHs [4,5].

DEAD-box RHs have been implicated in RNA synthesis, modification, cleavage, and degradation, as well as in ribosome biogenesis and translation initiation [4]. Moreover, they are also involved in the ATP-dependent rearrangement of intermolecular and intramolecular RNA structures or remodeling of ribonucleoprotein complexes [4,5,15]. In *Arabidopsis*, AtRH3, AtRH22/HS3, and AtRH39, for instance, encode chloroplastic DEAD-box RHs, and they are associated with RNA splicing and ribosomal RNA (rRNA) maturation [16,17,18,19]. AtRH38/LOS4 plays an essential role in the exporting of RNA from the nucleus to the cytoplasm [16,20]. AtRH14, AtRH20, AtRH30, and AtRH40 are involved in nonsense RNA decay and ribosome biogenesis [21]. In rice, OsRH2 and OsRH34 are core components of the exon junction complex, which play important roles in gene expression [22].

Despite their sequences and structural similarities, each DEAD-box RH is thought to have different functions in plant development [10,23]. In *Arabidopsis*, for instance, loss-of-function mutants of *AtRH3*, *AtRH22*/*HS3*, or *AtRH39* cause abnormal chloroplast development and pale-green seedling phenotypes [16,17,18,19]. *AtRH47*/*ISE1* affects embryonic development [24]; *AtRH36*/*SWA3* is required for the proper development of the female gametophyte [25,26]; and *AtRH57* responds to sugar involving glucose-mediated abscisic acid (ABA)-dependent inhibition of germination and seedling development [27]. In rice, *OsRH15*/*AIP2* and *OsRH56*/*AIP1* regulate programmed cell death during tapetum degeneration [28]. In addition, a number of DEAD-box RHs play important roles in plant abiotic stress tolerance via their functions in specific RNA processing events [29,30]. It has been demonstrated that *Arabidopsis AtRH38*/*LOS4* regulates the expression of DREB/CBFs under chilling stress [16,31]; *AtRH7* and *AtRH42*/*RCF1* are up-regulated under cold-stress conditions. In addition, *atrh7* and *atrh42*/*rcf1* mutants are more sensitive to cold stress than wild type (WT), whereas *AtRH42*/*RCF1* OXs are more tolerant to cold stress than WT [32,33]. The expression of *STRS1* and *STRS2* genes in *Arabidopsis* are rapidly down-regulated by various stresses and by ABA, and mutants exhibit a significant increase in salt-, osmotic-, and heat-stress tolerances, as well as enhanced expression of stress-inducible genes [34,35]. *AtRH5*, *AtRH9*, and *AtRH25*, three DEAD-box RHs in *Arabidopsis*, respond to multiple abiotic stresses [36]. In rice, *OsRH50*/*OsBIRH1* has been reported to function in modulating defense responses against pathogenic infections and oxidative stress [37]. Ectopic expression of *PDH45*, a DEAD-box RH in pea (*Pisum sativum*), improves salt tolerance in rice and tobacco (*Nicotiana tacum*), and ectopic expression of *PDH47*, another pea DEAD-box RH, enhances drought tolerance in rice [38,39].

In the present study, we used an activation tagging system to identify novel salt-stress-responsive genes in *Arabidopsis*. The activation tagging system makes it possible to identify genes which have a functional redundancy, or those for which loss-of-function mutants show lethality [40,41,42,43]. In addition, activated phenotypes can be observed directly without the generation of OXs [40,41,42,43]. We isolated *AtRH17*, a DEAD-box RH gene, using an activation tagging approach, and demonstrated that AtRH17 is involved in salt-stress tolerance as a nuclear protein in *Arabidopsis*.

## 2. Results

### 2.1. Isolation of AtRH17 via Activation Tagging

**Figure 1:**
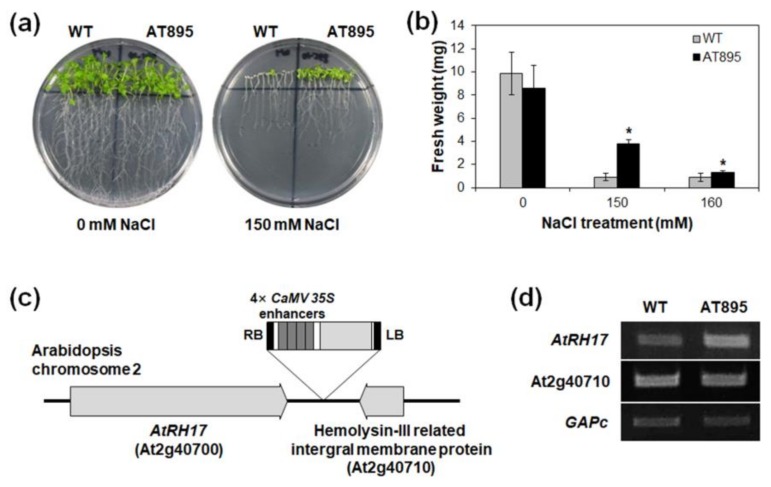
Isolation of the *AtRH17* gene involved in salt-stress tolerance from activation tagging line analysis. (**a**,**b**) Responses of wild type (WT) and AT895 seedlings to 0, 150, and 160 mM NaCl. Seven-day-old seedlings were transferred onto NaCl-containing Murashige and Skoog (MS) agar media, and photographs were taken seven days after NaCl treatment. Fresh weight (FW) was measured seven days after NaCl treatment. In (**b**), error bars represent standard deviation (*n* = 30 plants) and * indicate *t*-test *p* < 0.05. (**c**) Schematic map of the T-DNA insertion position in the genomic DNA of AT895. (**d**) Expression analysis of *AtRH17* (At2g40700) and hemolysin-III related integral membrane protein gene (At2g40710) in the AT895 plants by semi-quantitative reverse transcription (RT)-PCR. *GAPc* was used as an internal control.

To isolate novel salt-stress-responsive genes, we screened T_1_ activation tagging lines of *Arabidopsis* harboring pFGL942, an activation tagging vector containing four copies of *cauliflower mosaic virus* (*CaMV*) *35S* enhancers (Supplementary Figure S1a), as described previously [43]. Through primary and secondary screening using T_2_ plants, we isolated the AT895 line, which exhibited a salt-tolerant phenotype during germination (Supplementary Figure S1b,c). For further experiments, we isolated homozygotes of the AT895 line, and then analyzed the salt-stress response of the AT895 line at the seedling stage. To validate the isolated line, T_3_ seedlings of the AT895 line were transferred onto 0, 150, and 160 mM NaCl-containing Murashige and Skoog (MS) agar media. As a result, AT895 line seedlings were more tolerant and showed higher fresh weight (FW) than WT seedlings under 150 and 160 mM NaCl treatment conditions (Figure 1a,b), suggesting that the AT895 line is more tolerant to salt stress than WT during both germination and seedling development.

To identify the T-DNA tagging site in the AT895 line, we extracted genomic DNA from AT895 plants and performed a thermal asymmetric interlaced (TAIL)-PCR. Sequencing analysis using amplified PCR fragments revealed that *CaMV 35S* enhancers were inserted between At2g40700 and At2g40710 on *Arabidopsis* chromosome 2 (Figure 1c). At2g40700 encodes AtRH17, a DEAD-box RH, whereas At2g40710 encodes a hemolysin-III related integral membrane protein. We analyzed transcript levels of At2g40700 and At2g40710 in T_2_ plants of the AT895 line using semi-quantitative reverse transcription (RT)-PCR to identify a gene activated by *CaMV 35S* enhancers. This analysis revealed that At2g40700 transcription was higher in the AT895 line than WT, whereas no significant differences were detected in At2g40710 transcription between the AT895 line and WT (Figure 1d), implying that *AtRH17* is activated in the AT895 line.

### 2.2. Phylogenetic Analysis of AtRH17

**Figure 2:**
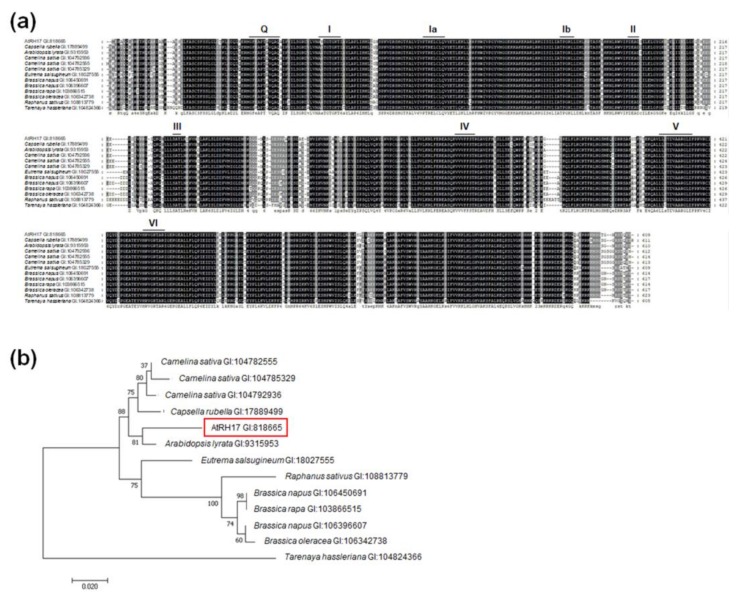
Conservation of DEAD-box RNA helicase (RH) motifs between AtRH17 and its 12 orthologs. (**a**) Multiple sequence alignment was carried out with amino acid sequences of the entire open reading frames (ORFs) of AtRH17 and the 12 orthologs using ClustalX 2.1 program. The gene ID number of each protein sequence is as follows: AtRH17: 818665; *Capsella rubella*: 17889499; *Arabidopsis lyrata*: 9315953; *Camelina sativa*: 104792936, 104782555, 104785329; *Eutrema salsugineum*: 18027555; *Brassica napus*: 106450691, 106396607; *Brassica rapa*: 103866515; *Brassica oleracea*: 106342738; *Raphanus sativus*: 108813779; *Tarenaya hassleriana*: 104824366. (**b**) Molecular phylogenetic tree of AtRH17 and the 12 orthologs was generated with the conserved regions including the well-conserved nine motifs (48–442 aa in AtRH17) using Maximum Likelihood method in MEGA 7.0.26 software. The number on each node indicates the bootstrap value for 1000 replicates.

DEAD-box RHs have been found in most prokaryotes and all eukaryotes, including plants [4,5,6]. To analyze conserved motifs in AtRH17, we performed multiple alignments of AtRH17 and its 12 orthologs in nine species exhibiting high similarity with AtRH17 using amino acid sequences of the entire open reading frame (ORF), and found that all 13 proteins possessed the well-conserved nine motifs characteristic of DEAD-box RHs, such as Q, I, Ia, Ib, and II-VI (Figure 2a). In addition, BlastP analysis using the entire ORF of AtRH17 showed no paralog of AtRH17 among the other 57 DEAD-box RHs present in *Arabidopsis* (data not shown).

We generated a phylogenetic tree to compare the phylogenetic relationship between AtRH17 and the 12 orthologs. The alignment of the conserved regions, including the well-conserved nine motifs (48–442 aa in AtRH17), revealed that AtRH17 is the most similar to the DEAD-box RH gene found in *A. lyrata* (Figure 2b).

### 2.3. Subcellular Localization of AtRH17 in the Nucleus

**Figure 3:**
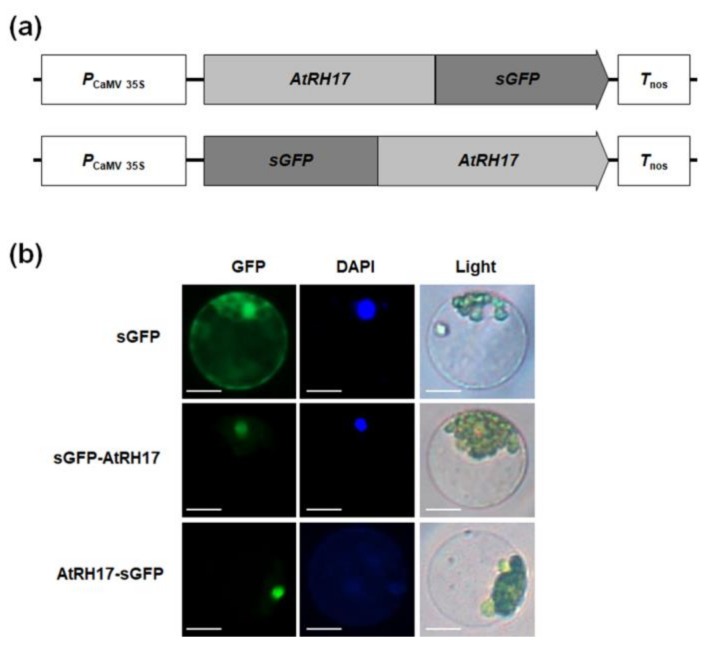
Investigation of the subcellular localization of AtRH17 in *Arabidopsis* protoplasts. (**a**) Schematic maps of the entire ORF of AtRH17 constructs fused to C-terminal or N-terminal synthetic green fluorescent protein (sGFP). (**b**) The subcellular localization of AtRH17 protein was examined by transient expression in Arabidopsis protoplasts. Left, GFP signal; middle, 4′,6-diamidino-2-phenylindole (DAPI) staining; right, light microscopic picture. Scale bars represent 10 μm.

To investigate the subcellular localization of AtRH17, we transformed *Arabidopsis* protoplasts with synthetic green fluorescent protein (sGFP)-AtRH17 and AtRH17-sGFP fusion constructs (Figure 3a). We observed that the GFP signals of both sGFP-AtRH17 and AtRH17-sGFP constructs were in the nucleus, in which the GFP signals overlapped with 4′,6-diamidino-2-phenylindole (DAPI) signals (Figure 3b), indicating that AtRH17 functions in the nucleus.

### 2.4. Expression of AtRH17 Is Unaffected by Osmotic Stress Conditions

**Figure 4:**
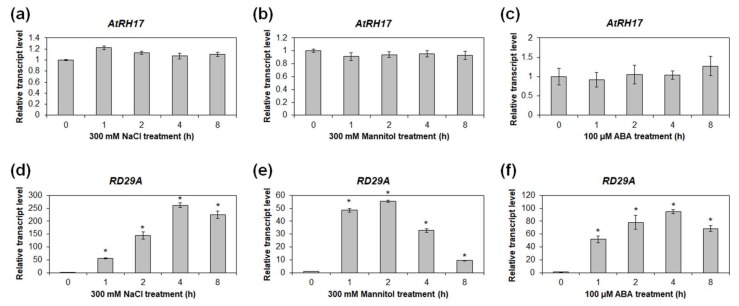
Expression analysis of *AtRH17* under osmotic-stress conditions. (**a**,**d**) Quantitative RT-PCR analysis of *AtRH17* (**a**) and *RD29A* (**d**) under 300 mM NaCl treatment for 0, 1, 2, 4, and 8 h. (**b**,**e**) Quantitative RT-PCR analysis of *AtRH17* (**b**) and *RD29A* (**e**) under 300 mM mannitol treatment for 0, 1, 2, 4, and 8 h. (**c**,**f**) Quantitative RT-PCR analysis of *AtRH17* (**c**) and *RD29A* (**f**) under 100 μM abscisic acid (ABA) treatment for 0, 1, 2, 4, and 8 h. *RD29A* was used as a control for NaCl, mannitol, and ABA treatments. *GAPc* was used as an internal control. Transcript levels at 0 h were set as 1. Three independent reactions were performed for each technical replicate. Two technical replicates were performed for each biological replicate. At least two biological replicates showed similar results, with one shown here. Error bars represent standard deviation (*n* = 6 reactions) and * indicate *t*-test *p* < 0.05.

Because the salt tolerance of the AT895 line strongly suggested that *AtRH17* could be related to osmotic stress response in *Arabidopsis*, we examined the expression patterns of *AtRH17* under osmotic-stress conditions, including NaCl, mannitol, and ABA treatments. Quantitative RT-PCR analysis using 10-day-old WT seedlings treated with 300 mM NaCl, 300 mM mannitol, and 100 μM ABA revealed that transcript levels of *AtRH17* were not significantly different after NaCl, mannitol, and ABA treatments compared with stress-free conditions (Figure 4a–c). The proper treatments of NaCl, mannitol, and ABA were confirmed by *RD29A* expression (Figure 4d–f). Semi-quantitative RT-PCR analysis produced similar results, in which *AtRH17* exhibited similar transcript levels before and after the NaCl, mannitol, and ABA treatments (Supplementary Figure S2).

### 2.5. Expression of AtRH17 during Developmental Stages and in Organs

**Figure 5:**
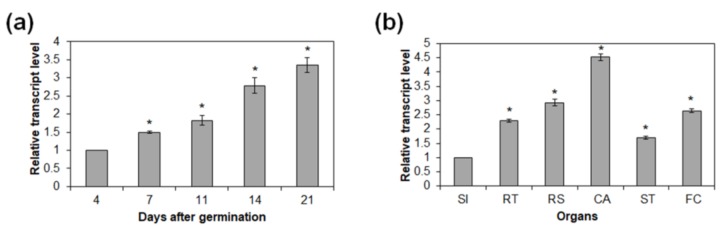
Temporal and spatial expression patterns of *AtRH17*. (**a**) Quantitative RT-PCR analysis of *AtRH17* in 4-, 7-, 11-, 14-, and 21-day-old WT seedlings grown under short-day (SD) conditions. *GAPc* was used as an internal control. Transcript level at 4 days after germination (DAG) was set as 1. (**b**) Quantitative RT-PCR analysis of *AtRH17* expression in organs of 36-day-old mature WT grown under long-day (LD) conditions. *GAPc* was used as an internal control. Transcript level in SI was set as 1. SI, siliques; RT, roots; RS, rosette leaves; CA, cauline leaves; ST, stems; FC, floral clusters. Three independent reactions were performed for each technical replicate. Two technical replicates were performed for each biological replicate. At least two biological replicates showed similar results, with one shown here. Error bars represent standard deviation (*n* = 6 reactions) and * indicate *t*-test *p* < 0.05.

Spatial and temporal expression patterns of *AtRH17* were investigated in *Arabidopsis* by quantitative RT-PCR. Transcript levels of *AtRH17* increased as plant developed from 4 to 21 days after germination (DAG) (Figure 5a). In mature plants, *AtRH17* transcription was highest in cauline leaves compared to other organs examined, such as siliques, roots, rosette leaves, stems, and floral clusters (Figure 5b). Semi-quantitative RT-PCR analysis showed similar results to those of the quantitative RT-PCR analysis (Supplementary Figure S3).

### 2.6. AtRH17 OXs Exhibit Tolerance to Salt Stresses, but Not to Mannitol and Freezing Stresses

**Figure 6:**
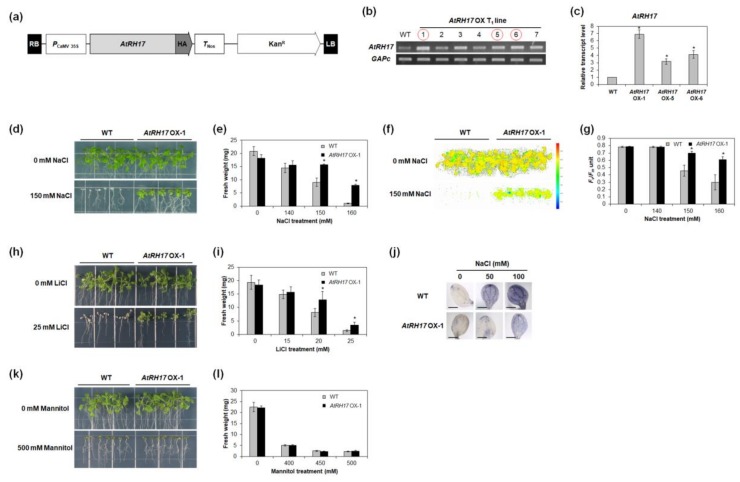
Osmotic-stress response of *AtRH17*-overexpressing transgenic plant (OX) seedlings. (**a**) Schematic map of vector for overexpression of *AtRH17*. (**b**) Selection of T_1_ lines overexpressing *AtRH17* by semi-quantitative RT-PCR analysis. Circled lines were selected for further analysis. *GAPc* was used as an internal control. (**c**) The transcript levels of *AtRH17* in *AtRH17* OXs were determined by quantitative RT-PCR analysis. *GAPc* was used as an internal control. Transcript level in WT was set as 1. Three independent reactions were performed for each technical replicate. Two technical replicates were performed for each biological replicate. Error bars represent standard deviation (*n* = 6 reactions) and * indicate *t*-test *p* < 0.05. (**d**) Responses of WT and *AtRH17* OX-1 T_3_ seedlings to 0, 140, 150, and 160 mM NaCl. Five-day-old seedlings were transferred onto NaCl-containing MS agar media and photographs were taken 15 days after NaCl treatments. (**e**) FW was measured 15 days after NaCl treatments. (**f**) Fluorescent image of Photosystem (PS) II activity (*F_v_*/*F_m_*) was taken 15 days after NaCl treatments. (**g**) PS II activity (*F_v_*/*F_m_*) was measured 15 days after NaCl treatments using FluorCam. (**h**) Responses of WT and *AtRH17* OX-1 T_3_ seedlings to 0, 15, 20, and 25 mM LiCl. Five-day-old seedlings were transferred onto LiCl-containing MS agar media and photographs were taken 15 days after LiCl treatments. (**i**) FW was measured 15 days after LiCl treatments. (**j**) Superoxide accumulations in cotyledons of 7-day-old WT and *AtRH17* OX-1 seedlings were detected by nitro blue tetrazolium (NBT) staining after 2 h NaCl treatments. Scale bars represent 2 mm. (**k**) Responses of WT and *AtRH17* OX-1 T_3_ seedlings to 0, 400, 450, and 500 mM mannitol. Five-day-old seedlings were transferred onto mannitol-containing MS agar media and photographs were taken 16 days after mannitol treatments. (**l**) FW was measured 16 days after mannitol treatments. In (**e**,**g**,**i**,**l**), error bars represent standard deviation (*n* = 35 plants) and * indicate *t*-test *p* < 0.05. Three independent T_1_ lines showed similar results, with one shown here.

To analyze the functional roles of *AtRH17* in abiotic stress response, we generated *AtRH17* OXs (Figure 6a) and selected three independent T_1_ lines that had higher levels of *AtRH17* expression than WT (Figure 6b,c). Similar to the AT895 line, *AtRH17* OX seedlings showed more tolerant phenotypes, with higher FW than WT seedlings under 150 and 160 mM NaCl treatments (Figure 6d,e and Supplementary Figure S4). Evaluations of Photosystem II (PS II) activity are commonly used to examine the physiology of plants under salt-stress conditions [44]. We measured PS II activity, represented by *F_v_*/*F_m_*, in both *AtRH17* OX and WT plants. Under 150 and 160 mM NaCl treatments, *F_v_*/*F_m_* levels were significantly higher in *AtRH17* OXs than in WT (Figure 6f,g). Moreover, *AtRH17* OX seedlings displayed greater tolerance than WT under 20 and 25 mM LiCl treatments (Figure 6h,i). In addition, we measured the production of superoxide in *AtRH17* OXs under stress conditions because reactive oxygen species (ROS) production is usually enhanced in plants experiencing abiotic stresses [45], and found that superoxide production was significantly lower in *AtRH17* OXs than in WT under 50 and 100 mM NaCl treatments (Figure 6j), suggesting that the lower superoxide accumulation in *AtRH17* OXs compared to WT might be a contributing factor to the greater salt-stress tolerance displayed by *AtRH17* OXs.

Because salt stress typically results in osmotic stress [46], we assessed the response of *AtRH17* OXs to mannitol treatment, another source of osmotic stress, and found that there were no significant differences between *AtRH17* OX and WT seedlings under 400, 450, and 500 mM mannitol conditions (Figure 6k,l). We also examined the cold-stress response of *AtRH17* OXs. Three-week-old *AtRH17* OXs and WT on MS agar media were kept at −8 °C. No significant differences were detected between *AtRH17* OX and WT seedlings following freezing treatment (Supplementary Figure S5). These results indicated that *AtRH17* OXs are no more tolerant to mannitol and freezing stresses than WT.

**Figure 7:**
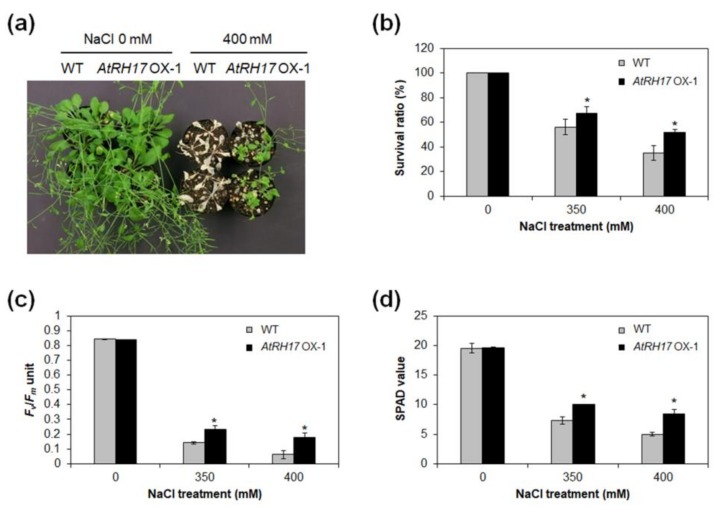
Salt-stress response of *AtRH17* OX mature plants. (**a**) Three-week-old WT and *AtRH17* OX-1 plants were watered with 0, 350, and 400 mM NaCl. Photograph was taken after 20-day NaCl treatments. (**b**) Survival ratio of WT and *AtRH17* OX-1 treated with 0, 350, and 400 mM NaCl for 20 days. (**c**) PS II activity (*F_v_*/*F_m_*) of WT and *AtRH17* OX-1 treated with 0, 350, and 400 mM NaCl for 17 days. (**d**) SPAD values of WT and *AtRH17* OX-1 treated with 0, 350, and 400 mM NaCl for 17 days. Error bars represent standard deviation (*n* = 30 plants) and * indicate *t*-test *p* < 0.05. Three independent T_1_ lines showed similar results, with one shown here.

Next, we investigated the salt-stress response of *AtRH17* OXs at mature plants stage. To this end, 3-week-old *AtRH17* OXs and WT were treated with 0, 350, and 400 mM NaCl solutions for three weeks. We then determined the survival ratio, PS II activity, and chlorophyll contents of *AtRH17* OXs and WT under both NaCl-free conditions and following NaCl treatments. No significant differences were detected between *AtRH17* OXs and WT under NaCl-free conditions, but *AtRH17* OXs were more tolerant and had higher survival ratios than WT in the 350 and 400 mM NaCl treatments (Figure 7a,b). The PS II activity assessed by *F_v_*/*F_m_* was also higher in *AtRH17* OXs than WT in the 350 and 400 mM NaCl treatments (Figure 7c). In addition, higher chlorophyll contents, as represented by SPAD values, were detected in *AtRH17* OXs than WT in the 350 and 400 mM NaCl treatments (Figure 7d), demonstrating that mature *AtRH17* OXs are more tolerant than WT under salt-stress conditions. Moreover, to confirm the osmotic-stress response of *AtRH17* OXs at mature plant stage, we treated drought stress to mature *AtRH17* OXs, and then determined the survival ratio, PS II activity, and chlorophyll contents of *AtRH17* OXs and WT under both drought-free conditions, and following drought treatments. No significant differences were detected between *AtRH17* OXs and WT under both drought-free conditions and following drought treatments (Supplementary Figure S6). Taken together, our results suggested that overexpression of *AtRH17* confers tolerance to salt stress specifically, and at both the seedling and mature plant stages.

### 2.7. Expression of ABA-Dependent and ABA-Independent Salt-Stress-Responsive Genes Does Not Alter, but is Lower in AtRH17 OXs under Salt Stress Conditions

**Figure 8:**
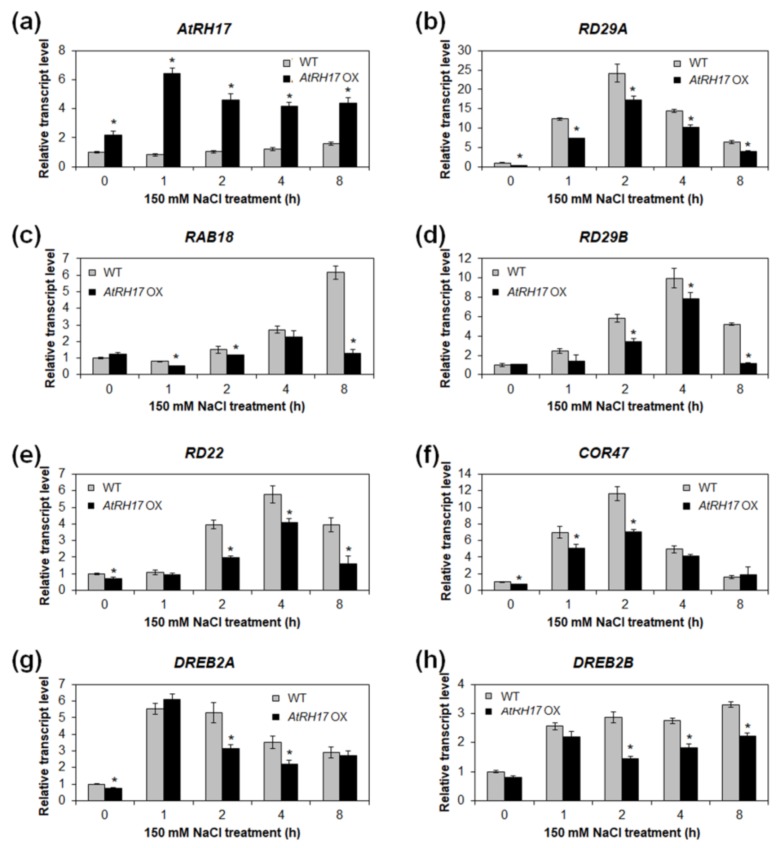
Expression patterns of ABA-dependent and ABA-independent salt-stress-responsive genes in *AtRH17* OXs. Quantitative RT-PCR analysis of *AtRH17* (**a**), *RD29A* (**b**), *RAB18* (**c**), *RD29B* (**d**), *RD22* (**e**), *COR47* (**f**), *DREB2A* (**g**), and *DREB2B* (**h**) in WT and *AtRH17* OX seedlings under 150 mM NaCl treatment for 0, 1, 2, 4, and 8 h. *GAPc* was used as an internal control. Transcript levels at 0 h in WT were set as 1. Three independent reactions were performed for each technical replicate. Two technical replicates were performed for each biological replicate. At least two biological replicates showed similar results, with one shown here. Error bars represent standard deviation (*n* = 6 reactions) and * indicate *t*-test *p* < 0.05.

Plant response to salt stress is usually mediated by ABA-dependent and/or ABA-independent signaling pathways [47]. To verify whether *AtRH17* is involved in ABA-dependent or ABA-independent salt-stress signal pathways, we examined the expression of ABA-dependent and ABA-independent salt-stress-responsive genes in *AtRH17* OXs under salt-stress conditions. Quantitative RT-PCR analysis using 10-day-old seedlings treated with 150 mM NaCl revealed that the expression of *AtRH17* was increased in *AtRH17* OXs after NaCl treatment, whereas the expression of *AtRH17* was not significantly changed in WT after NaCl treatment (Figure 8a). Expression of all the ABA-dependent or ABA-independent salt-stress-responsive genes, such as *RD29A*, *RAB18*, *RD29B*, *RD22*, *COR47*, *DREB2A*, and *DREB2B*, were elevated in *AtRH17* OXs under NaCl treatment. Notably, the transcription levels of all those genes were lower in *AtRH17* OXs than WT after NaCl treatment (Figure 8b–h), suggesting that overexpressed *AtRH17* inhibits the increase of those stress-responsive genes’ transcriptions. Our results indicated that salt-stress tolerance of *AtRH17* OXs is mediated by a pathway or mechanism other than the well-known ABA-dependent and ABA-independent stress-responsive pathways.

## 3. Discussion

Activation tagging is useful for isolating novel stress-responsive genes, and makes it possible to study genes that have functional redundancy or for which loss-of-function mutants demonstrate lethality [40,41,42,43]. In the present study, we isolated *AtRH17*, an *Arabidopsis* DEAD-box RH gene, involved in salt-stress response, using an activation tagging system. DEAD-box RHs have nine conserved motifs with the D-E-A-D sequence in motif II [3,8,9,10], and they have functional roles in RNA synthesis and processing, ribosome biogenesis, the initiation of translation, and the regulation of riboprotein complexes [4,8,48]. AtRH17 has orthologs in other plant species with well-conserved nine motifs (Figure 2a), but, interestingly, it does not have a paralog, indicating that AtRH17 may have unique role in *Arabidopsis*. Because *AtRH17* transcription does not increase under salt treatment, it might be very difficult to isolate *AtRH17* by expression analysis such as microarray or RNA-seq, demonstrating that the activation tagging system is useful method to isolate *AtRH17* as a salt-stress-responsive gene. We have previously isolated *S-RBP11*, a small RNA-binding protein gene, and *AtSFT12*, a Qc-SNARE protein gene, using an activation tagging system [43,49].

The activation tagging line, AT895, in which *AtRH17* is activated, was more tolerant to high salt stress than WT during germination and seedling development (Supplementary Figure S1 and Figure 1). In addition, *AtRH17* OXs exhibited greater tolerance than WT under salt-stress conditions at both the seedling and mature plant stages (Figure 6 and Figure 7). Superoxide accumulation was lower in *AtRH17* OXs than WT (Figure 6j), which possibly contributes to the higher salt-stress tolerance of *AtRH17* OXs. Moreover, *AtRH17* OXs were not significantly more tolerant than WT under mannitol, freezing, and drought treatments (Figure 6 and Supplementary Figures S5 and S6), implying that *AtRH17* is involved in the salt-stress response, not in osmotic- and cold-stress responses. These results indicated that the ectopic overexpression of *AtRH17* contributes to salt-stress tolerance during most *Arabidopsis* vegetative development stages, and that *AtRH17* is involved specifically in the salt-stress response.

Assessment of *AtRH17* expression under salt-stress and osmotic-stress conditions revealed that *AtRH17* transcription was unchanged by salt treatment, nor by mannitol and ABA treatments (Figure 4 and Supplementary Figure S2). In addition, we found that *AtRH17* expression increased as *Arabidopsis* seedlings developed (Figure 5). Because the elevated transcript level of *AtRH17* in OXs can confer elevated salt tolerance, these results suggested that *AtRH17* potentially contributes to salt tolerance in a gradual manner as *Arabidopsis* seedlings develop.

The results of the expression analysis of ABA-dependent and ABA-independent salt-stress-responsive genes indicated that the expression of all of the ABA-dependent and ABA-independent salt-stress-responsive genes included in our analysis (*RD29A*, *RAB18*, *RD29B*, *RD22*, *COR47*, *DREB2A*, and *DREB2B*) were no higher in *AtRH17* OXs than WT under salt-stress conditions; on the contrary, the expression levels of all of these genes were lower in *AtRH17* OXs than WT (Figure 8), indicating that AtRH17 might function as a negative regulator of expression of those genes, and that the salt-stress tolerance of *AtRH17* OXs might be mediated by some other pathway than the well-known ABA-dependent or ABA-independent stress-responsive pathways.

We determined AtRH17 to be localized in the nucleus (Figure 7). Previous research has shown that various nuclear DEAD-box RHs play functional roles in pre-rRNA processing [27], ribosome biogenesis [13], messenger RNA (mRNA) exporting [16], and RNA-directed DNA methylation [35]. In *Arabidopsis*, STRS1 and STRS2 are involved in epigenetic gene silencing, resulting in suppression of abiotic-stress-responsive genes [34,35]. Mutants of *STRSs* are more tolerant to salt, osmotic, and heat stresses, whereas *STRSs* OXs are more sensitive [34,35]. AtRH7/PRH75 participates in cold tolerance via regulation of CBF genes through involvement in rRNA biogenesis [32]; as such, it is possible that AtRH17 is also involved in RNA metabolism in the nucleus that regulates expression of salt-stress-responsive genes. Further studies exploring the specific molecular functions of AtRH17 are necessary.

Taken together, our results demonstrated that *AtRH17*, encoding a nuclear DEAD-box RH, is involved in salt-stress tolerance in *Arabidopsis*.

## 4. Materials and Methods

### 4.1. Plant Materials and Growth Conditions

All *Arabidopsis* lines used in this study were of the ecotype Columbia (Col-0). Seeds were surface-sterilized and germinated on MS agar media, as described previously [50]. Seedlings were grown under short-day (SD) (8 h light/16 h dark) or long-day (LD) (16 h light/8 h dark) photoperiods, with temperature maintained at 22 °C.

### 4.2. Plasmid Construction and Plant Transformation

To generate a vector for *AtRH17* OXs, an entire ORF of *AtRH17* was cloned into pFGL1400, in which the modified *CaMV 35S* promoter directs the constitutive expression of *AtRH17* in frame following hemagglutinin (HA) [50].

For protein subcellular localization, the entire ORF of *AtRH17* was cloned into the binary vectors pFGL1283 and pFGL1292 under the control of a modified *CaMV 35S* promoter, in frame with N-terminal and C-terminal sGFP, respectively. Primers used for PCR are listed in Supplementary Table S1.

All constructs were introduced into *Agrobacterium tumefaciens* strain GV3101 using the freeze-thaw method [51] and subsequently transformed into *Arabidopsis* plants using the floral-dipping method [52]. Transgenic plants were selected on MS agar media with kanamycin (50 μg/mL). T_3_ or T_4_ homozygous lines were used for subsequent analysis.

### 4.3. Activation Tagging Line Screening and Plant Stress Treatments

Procedures for activation tagging line screening followed those described previously [43].

For RT-PCR analysis under osmotic stress conditions, 10-day-old WT seedlings grown under SD conditions were exposed to filter papers soaked with MS solution containing 300 mM NaCl, 300 mM mannitol, and 100 μM ABA. The seedlings were harvested separately after 1, 2, 4, and 8 h, with 0 h as the control. For the analysis of salt-stress-responsive gene expression under salt-stress conditions, WT and *AtRH17* OX seedlings grown under SD conditions were exposed to filter papers soaked with MS solution containing 150 mM NaCl. Seedlings were harvested separately after 1, 2, 4, and 8 h, with 0 h as the control.

To observe seedling growth under osmotic-stress conditions, seeds were sown and grown on pure MS agar media for five days. For NaCl treatment, fully germinated seedlings were then transplanted to MS agar media supplemented with NaCl at concentrations of 0, 140, 150, and 160 mM, and FW and photosynthetic activity (*F_v_*/*F_m_*) of seedlings were measured after 10 or 15 days. For LiCl treatment, seedlings were transplanted to MS agar media containing LiCl at concentrations of 0, 15, 20, and 25 mM, and seedling FW was measured after 15 days. For mannitol treatment, seedlings were transplanted to MS agar media containing mannitol at concentrations of 0, 400, 450, and 500 mM, and seedling FW was measured 16 days after treatment. For freezing treatment, 3-week-old plants on MS agar media were kept at −8 °C for 0, 1, 2, and 4 h, and then allowed to recover for 5 days at 22 °C.

To evaluate the response of mature *AtRH17* OXs to NaCl exposure, 3-week-old plants cultivated under LD conditions were subjected to 0, 350, and 400 mM NaCl for three weeks, at 3–4-day intervals.

To assess the response of mature *AtRH17* OXs to drought stress, 3-week-old plants cultivated under LD conditions were withheld watering for 12 days and then rewatered for five days.

### 4.4. PS II Activity (F_v_/F_m_) and Chlorophyll Content Measurement

PS II activity (*F_v_*/*F_m_*) of seedlings grown under SD conditions was measured using a FluorCam FC-800 (Photon Systems Instruments, Drasov, Czech), in accordance with the manufacturer’s instructions.

PS II activity (*F_v_*/*F_m_*) in the third or fourth rosette leaves of 6-week-old plants grown under LD conditions was measured using a Handy PEA chlorophyll fluorimeter (Hansatech, King’s Lynn, UK), in accordance with the protocol described previously [50].

Measurement of chlorophyll content (SPAD value) in the fourth or fifth rosette leaves of 6-week-old plants grown under LD conditions was performed using an SPAD-502 plus chlorophyll meter (Konica Minolta, Inc., Tokyo, Japan), following the procedures described by Seok et al. [50].

### 4.5. Histochemical Staining of Superoxide Production

For histochemical staining of superoxide, 7-day-old seedlings grown under SD conditions were placed on filter papers soaked with 0, 50, and 100 mM NaCl for 2 h and incubated in 6 mM nitro blue tetrazolium (NBT) solution for 2 h [53]. Chlorophyll was then removed by immersing the seedlings in 95% (*v/v*) ethanol at 50 °C for 2 h.

### 4.6. RNA Extraction and RT-PCR Analysis

Total RNA was extracted from seedlings and different organs of mature plants using an RNAqueous RNA Isolation Kit (Life Technologies, Carlsbad, CA, USA), supplemented with Plant RNA Isolation Aid (Life Technologies, Carlsbad, CA, USA). First-strand complementary DNA (cDNA) synthesis, semi-quantitative RT-PCR, and quantitative RT-PCR were performed following the procedures described by Seok et al. [50]. The primers used for PCR are listed in Supplementary Table S1.

### 4.7. Multiple Sequence Alignment and Phylogenetic Analysis

Conserved amino acid sequences of AtRH17 and its orthologs were aligned using ClustalX 2.1 software and manually corrected. MEGA 7.0.26 software was used to generate the phylogenetic tree based on a Maximum Likelihood analysis with a bootstrap of 1000.

### 4.8. Subcellular Localization of the AtRH17-GFP Fusion Protein

To examine the subcellular localization of AtRH17 in the protoplast of *Arabidopsis*, polyethylene glycol-mediated protoplast transformations were performed as described previously [54].

### 4.9. Statistical Analysis

IBM SPSS Statistics software version 23 (IBM Corp., Armonk, NY, USA) was used for statistical analysis.

## Supplementary Materials

Supplementary materials can be found at http://www.mdpi.com/1422-0067/19/12/3777/s1.

## Abbreviations

ABA	Abscisic acid
AT	Activation tagging
*CaMV*	Cauliflower mosaic virus
DAG	Days after germination
LD	Long-day
MS	Murashige and Skoog
NBT	Nitro blue tetrazolium
OXs	Overexpressing transgenic plants
RH	RNA helicase
ROS	Reactive oxygen species
SD	Short-day
