# Supplementary material for: Overexpression of the DEAD-Box RNA Helicase Gene AtRH17 Confers Tolerance to Salt Stress in Arabidopsis
Source: Int J Mol Sci. 2018 Nov 28;19(12):3777. doi: 10.3390/ijms19123777 (PMC6321491; doi:10.3390/ijms19123777)
Supplement: Supplementary file 1 [file ijms-19-03777-s001.pdf]

ijms-390001

Supplementary data

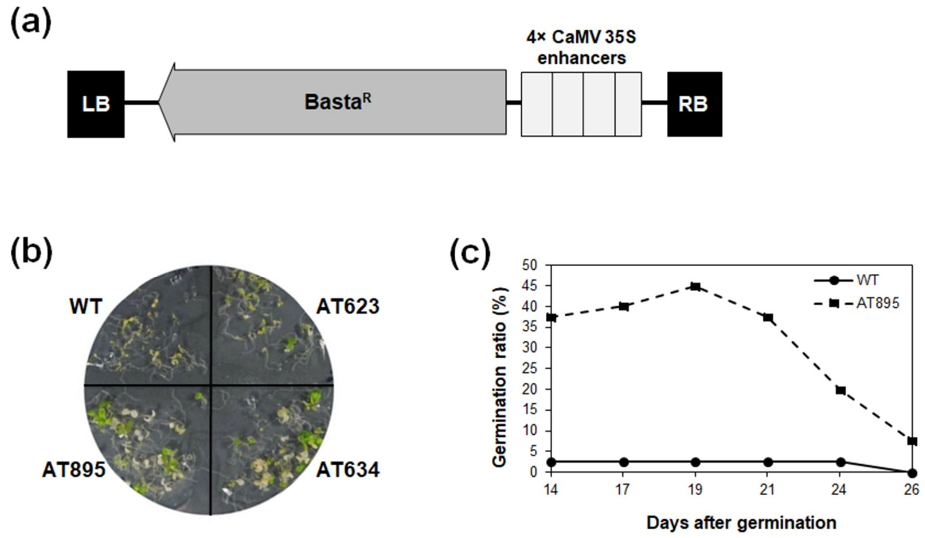

Figure S1. Isolation of AT895 from activation tagging lines. (**a**) Schematic map of the activation tagging vector, pFGL942. Four copies of *CaMV 35S* enhancers were used for the activation of genes. Basta-resistant gene was used as a selective marker for transgenic plants. (**b**) Germination of WT and T_2 plants of AT895, AT623, and AT634 on 210 mM NaCl-containing MS agar media. (**c**) Germination ratio of WT and T_2 plants of AT895 on 210 mM NaCl-containing MS agar media up to 26 DAG.

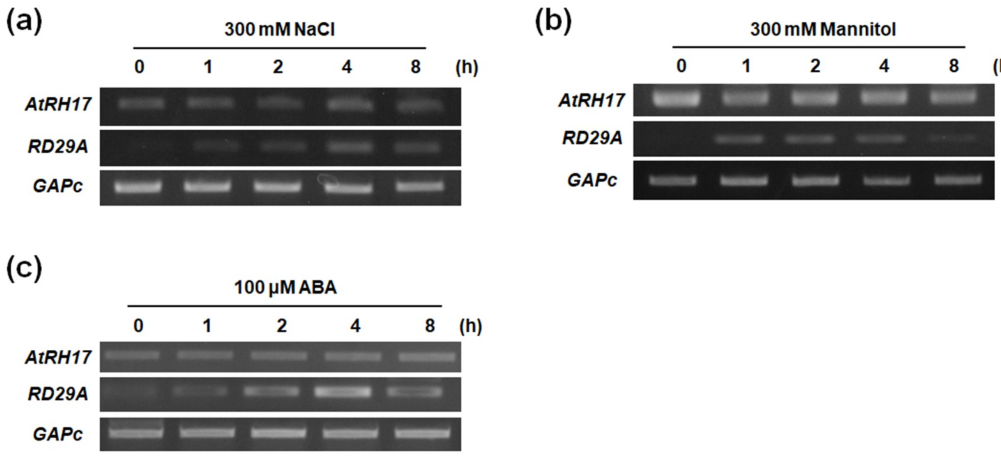

Figure S2. Expression analysis of *AtRH17* under osmotic stresses using semi-quantitative RT-PCR. (**a**) Expression of *AtRH17* under 300 mM NaCl treatment for 0, 1, 2, 4, and 8 hr. (**b**) Expression of *AtRH17* under 300 mM mannitol treatment for 0, 1, 2, 4, and 8 h. (**c**) Expression of *AtRH17* under 100 μM ABA treatment for 0, 1, 2, 4, and 8 hr. *GAPc* was used as an internal control. At least two biological replicates showed similar results, with one shown here.

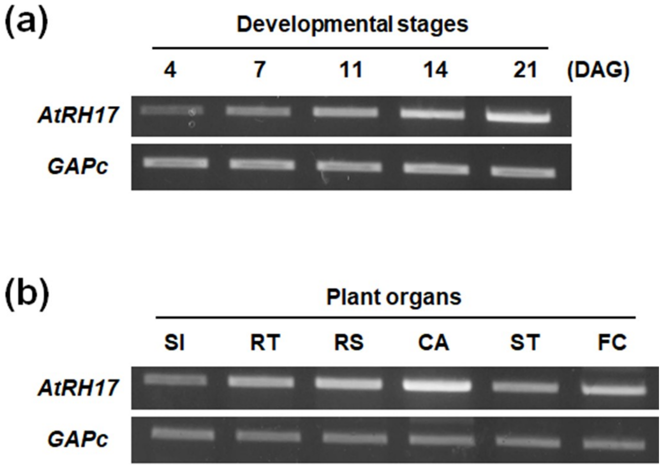

Figure S3. Analysis of temporal and spatial expression patterns of *AtRH17* using semi-quantitative RT-PCR. (**a**) Semi-quantitative RT-PCR analysis of *AtRH17* in 4-, 7-, 11-, 14-, and 21-day-old WT seedlings grown under SD conditions. *GAPc* was used as an internal control. (**b**) Semi-quantitative RT-PCR analysis of *AtRH17* expression in organs of 36-day-old WT grown under LD conditions. *GAPc* was used as an internal control. SI, siliques; RT, roots; RS, rosette leaves; CA, cauline leaves; ST, stems; FC, floral clusters. At least two biological replicates showed similar results, with one shown here.

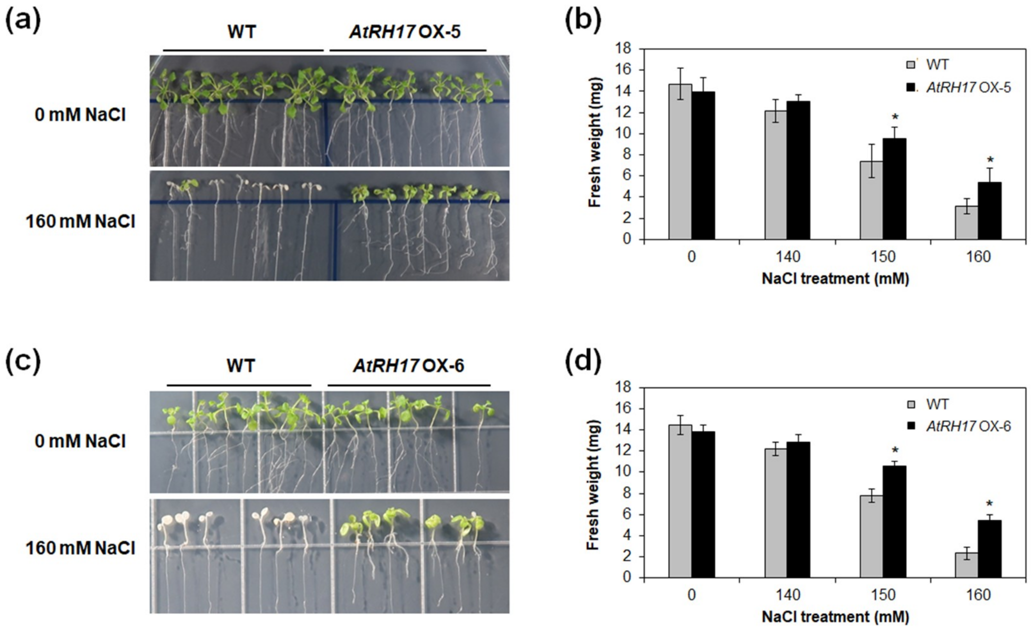

Figure S4. Salt-stress response of *AtRH17* OX seedlings. (**a**,**c**) Responses of WT and *AtRH17* OX T_3 seedlings to 0, 140, 150, and 160 mM NaCl. Five-day-old seedlings were transferred onto NaCl-containing MS agar media and photographs were taken 10 days after NaCl treatments. (**b**,**d**) FW was measured 10 days after NaCl treatments. Error bars represent the standard deviation (n = 35 plants) and * indicate t-test $P < 0.05$.

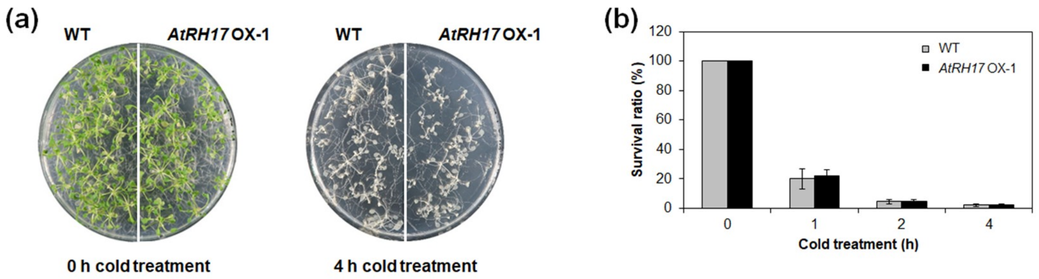

Figure S5. Cold-stress response of *AtRH17* OX seedlings. (**a**) Responses of WT and *AtRH17* OX-1 T_3 seedlings to freezing treatment for 0 and 4 hr. Three-week-old seedlings on MS agar media were kept at −8 °C, and photographs were taken after five days of recovery at 22 °C. (**b**) Survival ratio was measured after five days of recovery. Error bars represent standard deviation (n = 25 plants). Three independent T_1 lines showed similar results, with one shown here.

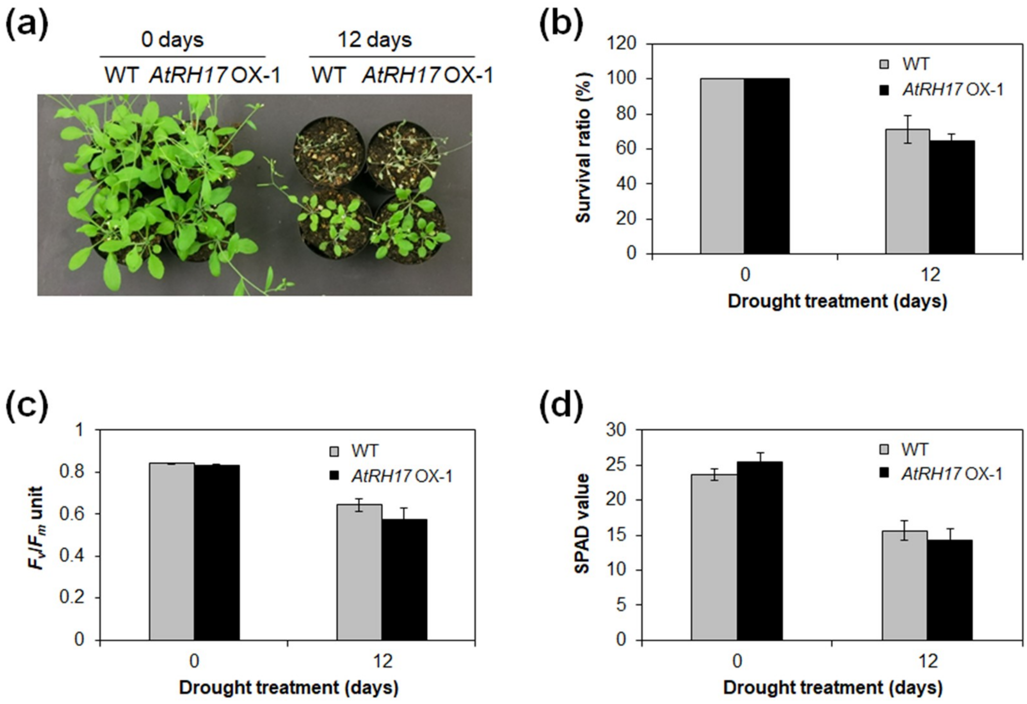

Figure S6. Drought-stress response of *AtRH17* OX mature plants. (**a**) Three-week-old WT and *AtRH17* OX-1 plants were dried for 12 days and then rewatered. Photograph was taken after five days of rewatering. (**b**) Survival ratio of WT and *AtRH17* OX-1 dried for 12 days and rewatered for five days. (**c**) PS II activity (F_v/F_m) of WT and *AtRH17* OX-1 dried for 12 days and rewatered for five days. (**d**) SPAD values of WT and *AtRH17* OX-1 dried for 12 days and rewatered for five days. Error bars represent standard deviation (n = 30 plants). Three independent T_1 lines showed similar results, with one shown here.

Table S1. List of primers used for PCR.

Gene	Forward	Reverse	Purpose
AtRH17	5′-TTCCCATACCCGGCAATATG-3′	5′-TCGGCGTAAGTAAGGGATTC -3′	Quantitative RT-PCR
GAPc	5′-GTGTCCCAACCGTTGATGTC-3′	5′-TCCCTTGAGTTTGCCTTCGG-3′	Quantitative RT-PCR
RD29A	5′-CCTGAAGTGATCGATGCACC-3′	5′-CAGTGGGTTTGGTGTAATCG-3′	Quantitative RT-PCR
RAB18	5′-TACCAGAACCGTCCAGGAGG-3′	5′-CGTACTCGTCATACTGCTGC-3′	Quantitative RT-PCR
RD29B	5′-TTCTTGGCTCGGTGGTAAAC-3′	5′-GGTGCCAAGTGATTGTGGAG-3′	Quantitative RT-PCR
RD22	5′-GTAAACCCGGTAAAAGAACC-3′	5′-TACACGAAAGGGTTTGCTCC-3′	Quantitative RT-PCR
COR47	5′-ATGTACCAGTTTCCACTACC-3′	5′-TCCTCTGCTTTCTCGTCGTG-3′	Quantitative RT-PCR
DREB2A	5′-GTGTTGCCAACGGTTCATAC-3′	5′-GAGGTATTCCGTAGTTGAGG-3′	Quantitative RT-PCR
DREB2B	5′-GAAGAGTCTTGTGGAACCAG-3′	5′-CCCAATACTGCTGCTCAAAC-3′	Quantitative RT-PCR
AtRH17	5'-TTCTGAGACAGAAGAGGAGG-3'	5'-TCGGCGTAAGTAAGGGATTC-3'	Semi-quantitative RT-PCR
GAPc	5'-CACTTGAAGGGTGGTGCCAAG-3'	5'-CCTGTTGTCGCCAACGAAGTC-3'	Semi-quantitative RT-PCR
RD29A	5'-GAAACAGAGTCTGCCGTGAC-3'	5'-TGCTGCCTTCTCGGTAGAGA-3'	Semi-quantitative RT-PCR
AtRH17 OX	5′-GTGGTCGACATGAAGAGAGCCCAACAATC-3′	5′-CGCGGATCCAGTTTTTTGTGTACTTCTAT-3′	Cloning
sGFP-AtRH17	5′-GTGGTCGACATGAAGAGAGCCCAACAATC-3′	5′-CGCGGATCCGAGTTTTTTGTGTACTTCTAT-3′	Cloning
AtRH17-sGFP	5′-GTGGTCGACATGAAGAGAGCCCAACAATC-3′	5′-CGCGGATCCTCAAGTTTTTTGTGTACTTC-3′	Cloning
